# The War Office and the Medical Profession

**Published:** 1917-09-22

**Authors:** 


### The Hospital

The Workers' Newspaper of Administrative Medicine and Institutional Life, Administration, National Insurance and Health.

No. 1633, Vol. LXII. SATURDAY, SEPTEMBER 22, 1917. PRICE ONE PENNY

### THE WAR OFFICE AND THE MEDICAL PROFESSION.

From the very commencement of the war we have without ceasing felt it to be a patriotic duty to promote in these columns the cordial co-operation of the medical profession with the authorities of the War Office. To the difficulties and responsibilities of the authorities we have ever extended a full recognition, and we have not hesitated to agree that in a time so exceptional as the present some sacrifice of civilian convenience and even of civilian rights may reasonably be demanded. In so far as the opportunity has been within our grasp we have endeavoured to lessen friction, to further mutual understanding, and to encourage the large patience and forbearance which are essential to the smooth working of great enterprises. This attitude, we are convinced, has commanded the general sympathy of the medical profession, as, indeed, may be plainly seen in the full and generous response which the profession has afforded to all the official calls for practical help and service. In entering a protest therefore against the recent action of the War Office in dismissing from the staffs of the military hospitals a considerable number of civilian practitioners who have during the last three years rendered most valuable service to our wounded soldiers, we cannot be accused of any hostile bias or of a desire to foster dissension. Our action is taken not only because the official proceeding inflicts an unjust slur upon individuals, but because, at least in our judgment, it involves prejudice to wounded men and is calculated to excite distrust and indignation in the general body of the profession.

The facts of the new situation are readily appreciated. In or near all the large centres of population military hospitals have been in existence since the beginning of the war. The work of these hospitals has only been possible by the active co-operation of a number of members of the local profession. Doubtless the professional skill and experience of these gentlemen have not an identical value, but it may be said with confidence that in the great majority of instances the work has been efficiently performed and much of it has been of a distinctly high order of excellence. More particularly, let it be noted, the practitioners concerned have now accumulated a special experience, and they may fairly be regarded as particularly well equipped and trained for their duties. Further, not a few of them have put aside some of their private interests in order the better to secure the welfare of their soldier patients. It is to these men that within the last few days has come an abrupt official announcement that their services are no longer required, accompanied by a strictly formal expression of gratitude for their past work. Can it be wondered that such an action has promoted widespread resentment and indignation? Some of the circumstances attending this action tend distinctly to aggravate its significance. In the first place members of the medical profession are not accustomed to a curt dismissal on a few days' notice, and, having answered an appeal, sometimes to their own personal loss, they are entitled to expect a due measure of courtesy. More important is the consideration that this proceeding of the War Office has not been discussed with the Medical War Committees which may reasonably be regarded as the authoritative representatives of the civilian profession. It is through the medium of these committees that the profession throughout the length and breadth of the country has been organised in such a fashion as to afford the maximum amount of assistance to the War Office, and this work has been accomplished without any expense to the Government. Yet it would seem that when the authorities conclude they can do without civilian help they cultivate a short way alike with committees and with individuals. There will, we are convinced, be a feeling of deep indignation at the official pushing to one side of organisations which have, in a spirit of unselfish patriotism, strained every effort to procure the medical assistance necessary for our armies. Once more, as is common knowledge, a committee has recently been appointed to investigate the organisation of the R.A.M.C., with a view to determine whether some economy of *personnel* may not be secured, and this committee, as a fact, has already entered on its task. Until the issue thus raised has been settled there would seem to be reason for claiming that no disturbance of the existing arrangements is justified, and that a scheme, which on the whole has worked well, ought to continue at least until the whole question has been passed under review. In short, the curt fashion of the dismissal, its presentation over the heads of the representatives of the civilian profession, and the knowledge that an inquiry on a related subject is still in progress, combine to mark the proceeding with the autocratic touch which, in this country at least, arouses resentment and some degree of suspicion.

To replace the dismissed practitioners it is alleged that medical graduates from the United States have been secured. We have nothing but a courteous welcome for our Transatlantic *confrères*, and this more particularly when the two nations are comrades in an enterprise of high moment to the welfare of humanity. But they, like the rest of us, have to attain efficiency in the school of experience, and we may without any discourtesy question whether, at least for some time, the judgment and skill of the newcomers can effectively replace the established craftsmanship which is the issue of some years of hard practical training. Certainly we are not going to encourage any technical objections or difficulties, but we cannot see without regret the loss from the service of our wounded fellow-countrymen of a body of experience which has been carefully gathered and effectively employed. In some quarters we note the suggestion is made that the new arrangement has for its motive the setting free of more practitioners for civilian practice. We doubt whether it will have any substantial value in this direction, and if its authors aspire to such an end, it is curious that before launching their scheme they did not consult the Medical War Committees, who alone have the information necessary to determine in advance how far this end will be furthered by the new development. Both by its form and by its substance this development is likely to promote much bitterness and resentment, and we think the general public as well as the profession has a right to a full explanation from the authors of it.

